# Quantifying DNA damage following light sheet and confocal imaging of the mammalian embryo

**DOI:** 10.1038/s41598-024-71443-x

**Published:** 2024-09-05

**Authors:** Darren J. X. Chow, Erik P. Schartner, Stella Corsetti, Avinash Upadhya, Josephine Morizet, Frank J. Gunn-Moore, Kylie R. Dunning, Kishan Dholakia

**Affiliations:** 1 Robinson Research Institute, School of Biomedicine, The University of Adelaide, Adelaide, Australia; 2 Institute for Photonics and Advanced Sensing, The University of Adelaide, Adelaide, Australia; 3 School of Biological Sciences, The University of Adelaide, Adelaide, Australia; 4 Centre of Light for Life, The University of Adelaide, Adelaide, Australia; 5 SUPA, School of Physics and Astronomy, University of St Andrews, North Haugh, St Andrews, Fife, UK; 6 School of Biology, University of St Andrews, North Haugh, St Andrews, Fife, UK

**Keywords:** Biological techniques, Optics and photonics, Physics

## Abstract

Embryo quality assessment by optical imaging is increasing in popularity. Among available optical techniques, light sheet microscopy has emerged as a superior alternative to confocal microscopy due to its geometry, enabling faster image acquisition with reduced photodamage to the sample. However, previous assessments of photodamage induced by imaging may have failed to measure more subtle impacts. In this study, we employed DNA damage as a sensitive indicator of photodamage. We use light sheet microscopy with excitation at a wavelength of 405 nm for imaging embryo autofluorescence and compare its performance to laser scanning confocal microscopy. At an equivalent signal-to-noise ratio for images acquired with both modalities, light sheet microscopy reduced image acquisition time by ten-fold, and did not induce DNA damage when compared to non-imaged embryos. In contrast, imaging with confocal microscopy led to significantly higher levels of DNA damage within embryos and had a higher photobleaching rate. Light sheet imaging is also capable of inducing DNA damage within the embryo but requires multiple cycles of volumetric imaging. Collectively, this study confirms that light sheet microscopy is faster and safer than confocal microscopy for imaging live embryos, indicating its potential as a label-free diagnostic for embryo quality.

## Introduction

Optical imaging has become ever more prevalent in the field of developmental biology^1,2^. This surge is primarily driven by its innate capacity to unravel intricate biological processes with high spatio-temporal resolution^3^. Historically, optical imaging has relied on the use of exogenous fluorescent labels to visualise and quantify specific biological phenomena within cellular structures. However, these external fluorescence labels have the potential to inadvertently perturb the underlying cell biology^4^ and pose challenges in the accurate interpretation of observed cellular changes. Recently, there has been a shift in the field towards label-free imaging^3^, specifically by capturing cellular autofluorescence. This approach leverages the inherent fluorescence emitted by endogenous molecules^2^, without the need for exogenous labels. Examples of this include specific co-factors associated with cellular metabolism^3^.

Label-free fluorescence microscopy holds great promise for the evaluation of early mammalian embryos. Current techniques for assessing embryo viability, such as visual inspection to evaluate preimplantation development or a biopsy of cells for genetic testing, are known to be highly subjective and inaccurate^5–7^. The inability to accurately predict embryo viability is a key contributor to the low success rate of clinical in vitro fertilisation (IVF; $\sim 30$% live birth rate per initiated cycle)^8^. Therefore, there is a need for an accurate and non-invasive diagnostic: optical imaging has the prospect of fulfilling this need. Recent studies, including our own work, have shown that label-free imaging analysis can detect metabolic differences that correlate with embryo viability^9–11^. This was performed with one-photon light sheet microscopy, epifluorescent or confocal microscopies. A point scanning approach has also been used in two-photon imaging^12^. Given the inherent simplicity of using a one-photon approach, making it amenable for future clinical translation, here we concentrate on a comparison between confocal and light sheet imaging, both in a one-photon embodiment. Our study determines the level of DNA damage accrued when acquiring a 3D volumetric image with both light sheet microscopy and confocal microscopy.

Confocal scanning microscopy has long been the gold standard for collecting cellular autofluorescence. For this technique, the entire sample volume is illuminated each time a z-plane is imaged^11^. Such an illumination scheme can lead to photobleaching and phototoxicity^13^. Nonetheless, confocal microscopy has continued to be used widely for studying developmental processes in various cell types, including human cornea^14,15^ and lung tissue^16^, as well as mouse and hamster embryonic cells^17–19^. Confocal microscopy has also been used for detecting dynamic changes in metabolism within live mouse oocytes^9^ and bovine embryos^20^. These studies employed a 405 nm light source to excite the endogenous metabolic fluorophore nicotinamide adenine dinucleotide phosphate (NAD(P)H)^21^. However, uncertainty surrounds the safety of this imaging modality for live embryos.

Light sheet microscopy has recently emerged as an alternative and prominent optical imaging modality for visualising and assessing embryo viability^10,22^. For such imaging, a thin sheet of light excites only the fluorescence from the z-plane of interest in the specimen. Therefore, this technique allows for rapid image acquisition. The approach has potentially reduced photobleaching when compared with epi-fluorescent imaging modalities, such as confocal microscopy. An example of such reduced photobleaching was seen in yeast cells tagged with exogenous fluorophores^23^. Overall, this has resulted in researchers using light sheet microscopy to study dynamic cellular processes such as embryo development and morphogenesis over a few hours to days, and in an array of species^10,24–37^. For example, we demonstrated the use of light sheet microscopy to non-invasively measure metabolic activity within mouse embryos^10^. This detected changes in the abundance of the metabolic fluorophores flavin adenine dinucleotide (FAD) and NAD(P)H throughout preimplantation development.

However, measurement of autofluorescence in either light sheet or confocal microscopy is not without its challenges: in particular, the signal may exhibit relatively weak intensity^4^. Autofluorescence is generally low compared to signals from exogenous fluorophores. A higher signal can generally be achieved by increasing excitation power, but typically at the cost of photobleaching and photodamage in the imaged sample^38^. Interestingly, light sheet microscopy achieves similar fluorescence signals to confocal microscopy with a lower light dose, thus reducing photobleaching and phototoxicity^39^. In this regard, Reynaud et al. showed that the photobleaching rate of exogenous fluorescent tags in yeast cells is six times higher with confocal microscopy than with light sheet imaging^23^. For the area of reproductive biology, prior investigations have demonstrated that embryos subjected to light sheet imaging did not impact their ability to complete preimplantation development^10,30^. However, such approaches do not provide a comprehensive investigation of photodamage, particularly DNA damage. The exposure of embryos to light during imaging can cause DNA damage^40^. Such damage is known to negatively impact embryos and is associated with poor developmental outcomes (decreased implantation potential/pregnancy success)^41,42^. Therefore, a robust assessment of DNA damage induced by light sheet and confocal imaging would be highly informative.

Here, we used both light sheet and confocal microscopy (at a wavelength of 405 nm) to image autofluorescence in blastocyst-stage embryos and determined the resultant impact on DNA integrity. For both modalities, we matched the signal-to-noise ratio of images and assessed DNA damage in the embryo. A sensitive assay for measuring DNA damage is immunohistochemistry for $\gamma$H2AX. Phosphorylation of the histone variant H2AX, forming $\gamma$H2AX, is indicative of double-strand breaks in DNA^43,44^. In our study, we utilised this assay to detect DNA damage in embryos following light sheet or confocal imaging. To the best of our knowledge, this marks the first confirmation that light sheet microscopy is a safe and preferred option for recording embryo autofluorescence.

## Results

### Characterisation of imaging with the light sheet microscopy and confocal microscopy

**Fig. 1:**
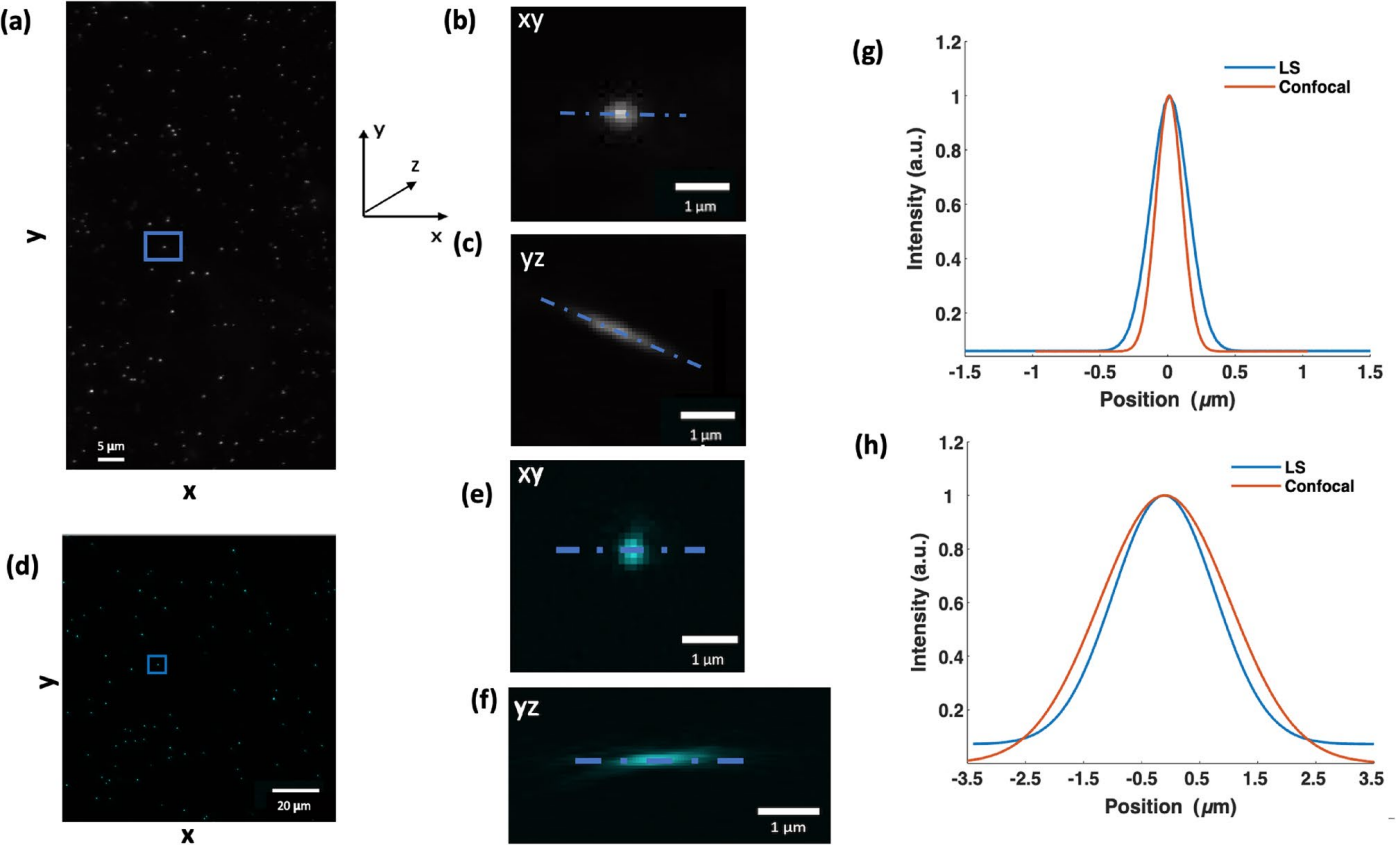
The light sheet (LS) propagates along the x-axis and is scanned with respect to the sample in the direction of the z-axis. (**a**) and (**d**) show the maximum intensity projections of images of 200 nm diameter fluorescent microspheres embedded in agarose acquired with the light sheet and the confocal setup, respectively. (**b**) and (**e**) are magnified views that show the single bead marked by the target in (**a**) and (**d**) in the *xy* plane. (**c**) and (**f**) are magnified views that show the intensity projection of the beads marked in (**a**) and (**d**) in the *yz* plane. In (**c**) the point-spread function is skewed due to the 45-degree illumination and detection angle with respect to the sample in the light sheet setup. (**g**) and (**h**) show the line profile across the beads marked by the targets and the line profile across the beads intensity projection in the *xy* and *yz* plane, respectively.

In order to compare the safety of confocal and light sheet microscopy, a preliminary characterisation of the two systems was performed. To determine the point spread function (PSF) of the light sheet and confocal microscope, we imaged 200 nm diameter isolated green fluorescent microspheres embedded in agarose. The maximum intensity projection of beads in the *xy* plane imaged using light sheet are shown in Fig. 1a. We determined the light sheet resolution by measuring the full width at half maximum (FWHM) in the lateral and axial directions from line profiles through twenty isolated fluorescent microspheres randomly sampled. For example, the maximum intensity profile for a single bead (marked by square target in Fig. 1a) in *xy* and *yz* planes are shown in Fig. 1b,c, respectively. The corresponding line profiles through the bead are represented in Fig. 1d,e, respectively. The lateral resolution was measured to be $0.656 \pm 0.06$ $\upmu$m. The axial resolution was $2.42 \pm 0.2$ $\upmu$m. The depth of field (DOF) of the light sheet itself was $\sim 70$ $\upmu$m. The DOF and light sheet amplitude on the screen ($\sim 2.5$ mm) determine the total field of view (FOV). As a result, we were able to image up to 20 embryos for a single z-stack. We used this DOF in our light sheet system to achieve a good axial resolution ($\sim 2.42$ $\upmu$m). If a propagation invariant beam such as an Airy beam was used, the same axial resolution can be achieved with an extended DOF^45^. An Airy beam can be readily incorporated into a light sheet geometry similar to the one we use, if desired^46^. Figure 1d shows the maximum intensity projections of 20 beads acquired using confocal microscopy. The maximum intensity profile for a single bead in *xy* and *yz* planes are shown in Fig. 1e,f, respectively. The corresponding line profiles through the bead (b,e and c,f) are represented in Fig. 1g,h, respectively. The lateral resolution was measured to be $0.439 \pm 0.016$ $\upmu$m. The axial resolution was $3.099 \pm 0.283$ $\upmu$m. We confirmed that our sample size (20 beads) fulfilled the criteria of a random sample (Q–Q plot and Shapiro–Wilk test; $P > 0.05$ for both lateral and axial data, respectively). We remark that we also performed a similar test on a smaller sample size of ten and recorded the same results ($P > 0.05$ for 20 vs. 10 beads: Lateral (0.774 vs. 0.716) and Axial (0.233 vs. 0.231).

**Fig. 2:**
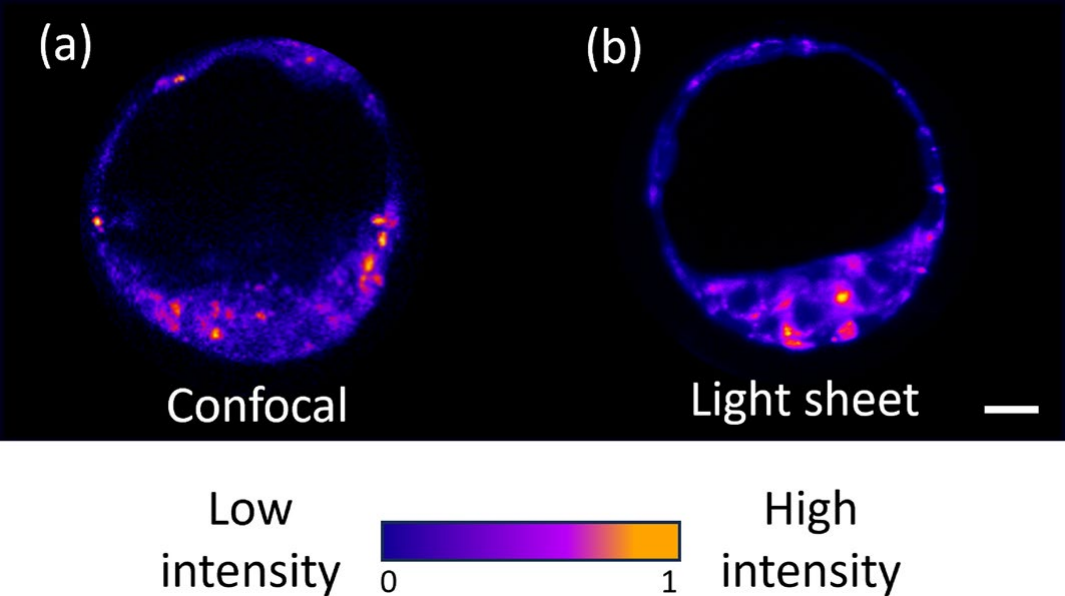
Representative images of autofluorescence signals recorded with excitation at a wavelength of 405 nm for blastocyst-stage embryos using (**a**) confocal (SNR = 15.7) or (**b**) light sheet (SNR=15.5) microscopy. The images displayed in (**a**) and (**b**) were derived from a single z-slice from the total collected z-planes. 3D reconstruction for embryos acquired on both imaging systems can be found in Supplementary File S2: Videos 1 and 2 for confocal and light sheet microscopy, respectively. Images have been cropped to a comparable scale with no sharpening or other post-processing applied. Scale bar = 20 $\upmu$m.

To achieve a faithful comparison between light sheet and confocal microscopy we standardised the parameters used for both imaging systems to provide a comparable signal-to-noise ratio (SNR) for embryo autofluorescence. Typically due to sample preparation and excitation laser conditions, fair comparisons between imaging systems can be complicated. To mitigate this, we examined the imaging parameters employed by each system, focusing on the SNR of autofluorescence signals recorded for a more robust comparison. To determine the SNR for confocal microscopy, we scanned the total volume of a single blastocyst-stage embryo ($\sim 100$ $\upmu$m in diameter). This took approximately thirty minutes. In contrast, the same volume was scanned with the light sheet microscope in approximately three minutes. Figure 2 shows representative images for a single z-plane of blastocyst-stage embryos, generated from their autofluorescence signal captured following confocal (Fig. 2a) or light sheet imaging (Fig. 2b). For each imaging system, we determined the SNR by measuring ten individual blastocyst-stage embryos with confocal and light sheet microscopy, which gives an average SNR value of $15.75 \pm 1.90$ and $15.45 \pm 3.45$, respectively (Fig. 2a,b). Supplementary File S2, and its associated videos, show 3D image reconstructions for autofluorescence of blastocyst-stage embryos obtained following imaging using confocal or light sheet microscopies. Interestingly, confocal microscopy had a limited imaging capability for this sample, resulting in a loss of autofluorescence signal in approximately half the volume of the embryo (Video 1; Supplementary File S2). In contrast, light sheet microscopy captured the entire volume of a blastocyst-stage embryo, allowing for true 3D image reconstruction (Video 2; Supplementary File S2). We postulate that the predominant contributing factor is photobleaching, an issue associated with the point-illumination approach.

### Effect of imaging with light sheet and confocal microscopy at a wavelength of 405 nm

**Fig. 3:**
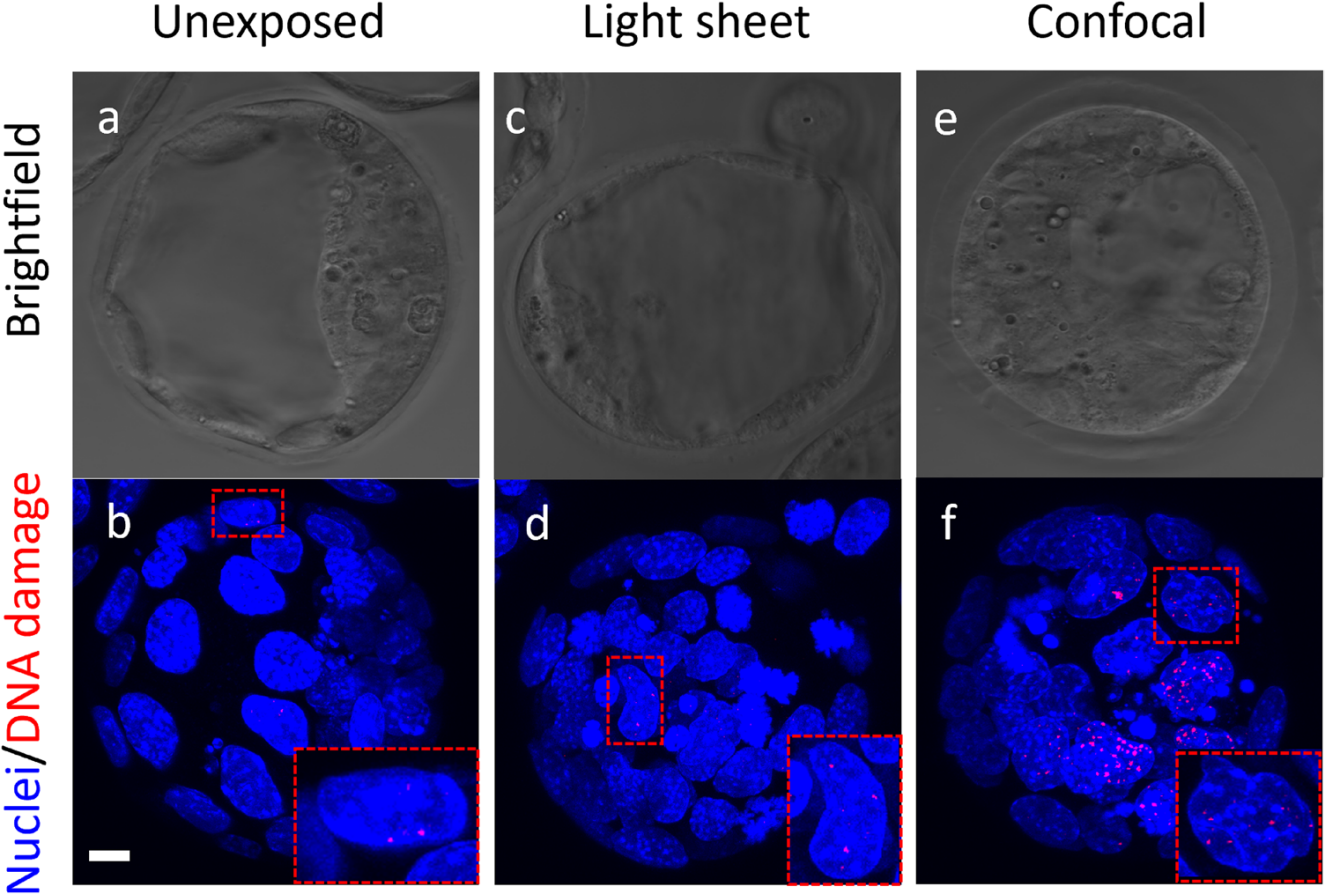
Representative images of DNA damage within embryos. Blastocyst-stage embryos were either not imaged (*Unexposed*; **a**, **b**), or imaged (at a wavelength of 405 nm) using light sheet microscopy (**c**,**d**) or confocal microscopy (**e**,**f**). Representative brightfield images of embryos (**a**,**c**,**e**) with corresponding maximum intensity projection of DNA damage (*red*: **b**,**d**,**f**) within individual nuclei (*blue*) are shown. Embryos were stained with DAPI (blue) and $\gamma$H2AX (red foci) to visualise individual cell nuclei and DNA double-stranded breaks, respectively (see insets). Scale bar = 20 $\upmu$m.

**Fig. 4:**
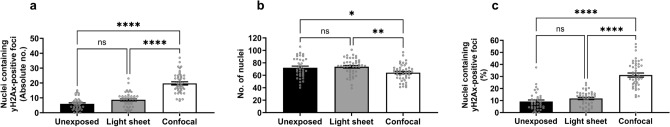
Imaging of embryos using confocal microscopy resulted in significantly fewer cells and higher levels of DNA damage. Blastocyst-stage embryos were kept in the dark (unexposed) or exposed to 405 nm laser excitation on either a light sheet or confocal microscope. Embryos were then returned to culture for 30 min prior to fixation. DNA damage and cell nuclei were identified via $\gamma$H2AX immunohistochemistry and DAPI staining, respectively. (**a**) shows the absolute number of nuclei containing $\gamma$H2AX-positive foci per embryo. (**b**) shows the total number of cells per embryo (DAPI-stained nuclei), while (**c**) expresses the levels of DNA damage as a proportion of nuclei containing $\gamma$H2AX-positive foci per embryo. Data are presented as mean ± SEM, n = 36–45 embryos per treatment group, from 3 independent experimental replicates, and analysed by one-way ANOVA with Tukey’s multiple comparison test (**a**,**b**) or Kruskal–Wallis with Dunn’s multiple comparison test (**c**). *$P <0.05$; **$P < 0.01$; ****$P < 0.0001$.

To evaluate the impact of imaging on embryo DNA integrity, blastocyst-stage embryos underwent $\gamma$H2AX staining following imaging with light-sheet or confocal microscopy (Fig. 3). Visually, embryos imaged using confocal microscopy appeared to have an increased number of nuclei containing $\gamma$H2AX-positive foci (red) when compared to unexposed (control) embryos (Fig. 3f,b, respectively). In contrast, embryos subjected to light sheet imaging appeared to have similar levels of DNA damage to unexposed embryos (Fig. 3d,b, respectively). These observations were confirmed following quantification of DNA damage (Fig. 4a). Specifically, embryos imaged with confocal microscopy had a significant (3.2-fold) increase in the number of nuclei containing $\gamma$H2AX foci when compared to the unexposed group (Fig. 4a; *Confocal* vs. *Unexposed*; $P < 0.0001$). No significant difference was observed between embryos that were not imaged and embryos that were imaged with light sheet microscopy (Fig. 4a; *Unexposed* vs. *Lightsheet*; $P > 0.05$). To confirm that the number of cells within an embryo did not confound our interpretation of imaging-induced DNA damage, we quantified cell number for each embryo (Fig. 4b) and presented DNA damage as a proportion of cells containing $\gamma$H2AX foci (Fig. 4c). Interestingly, we found that confocal imaging significantly reduced the number of cells within embryos when compared to the light sheet imaged and unexposed groups (Fig. 4b; $P < 0.05$), potentially indicating rapid cell depletion through light-induced apoptosis or or impaired proliferation^47^. However, this reduction in cell number did not alter the relationship between the three groups. When damage was presented as a proportion of nuclei containing $\gamma$H2AX foci; confocal imaged embryos had a significantly higher level of DNA damage when compared to light sheet imaged and unexposed embryos (Fig. 4c; $P < 0.0001$). Notably, embryos that were not imaged displayed a low level of DNA damage (Fig. 4c; *Unexposed*). This may be attributed to ambient light exposure from embryo handling during IVF, which does not occur during development in vivo^48,49^.

**Fig. 5:**
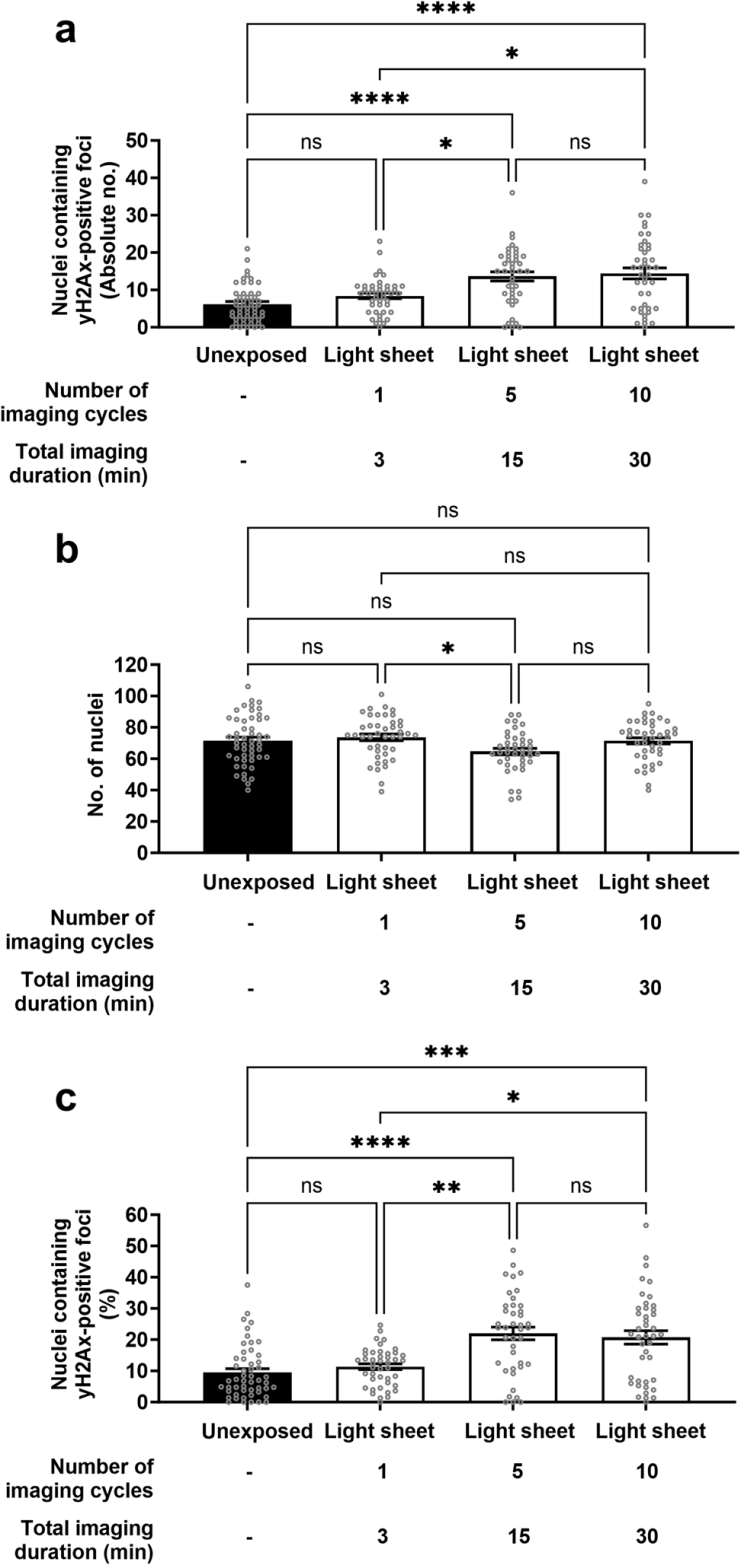
Increasing cycles of light sheet imaging induces increased levels of DNA damage. Blastocyst-stage embryos were either kept in the dark (unexposed), or subjected to increasing rounds of imaging using light sheet microscopy (one, five or ten z-stacks). The total imaging duration to acquire these z-stack(s) is shown. Following imaging, embryos were returned to the incubator for an additional 30 min prior to fixation. DNA damage and cell nuclei were identified via $\gamma$H2AX immunohistochemistry and DAPI staining, respectively. (**a**) shows the absolute number of nuclei containing $\gamma$H2AX-positive foci per embryo. (**b**) shows the total number of cells per embryo (DAPI-stained nuclei), while (**c**) expresses the levels of DNA damage as a proportion of nuclei containing $\gamma$H2AX-positive foci per embryo. Data are presented as mean ± SEM, n = 41–51 embryos, from 3 independent experimental replicates, analysed using Kruskal–Wallis with Dunn’s multiple comparison test. *$P < 0.05$; **$P < 0.01$; ***$P <0.001$; ****$P < 0.0001$.

Next, we examined whether multiple rounds of light sheet imaging resulted in elevated levels of DNA damage. This was evaluated by subjecting embryos to one, five or ten repeated cycles of volumetric imaging using light sheet microscopy (equivalent to a total imaging duration of three, fifteen or thirty minutes, respectively; Fig. 5). We found elevated levels of DNA damage with repeated cycles of imaging (Fig. 5; Light sheet: *one* vs. *five* and *ten* cycles; $P < 0.05$). Specifically, embryos imaged for five or ten cycles with light sheet microscopy had significantly higher levels of DNA damage when compared to embryos that were imaged once or not imaged (Fig. 5a; *five* or *ten* cycles vs. *Unexposed* or *one*; $P < 0.05$). As described above, we determined the impact of imaging on cell/nuclei number. Embryos that underwent five rounds of light sheet imaging had significantly fewer nuclei compared to embryos that were imaged once (Fig. 5b; $P < 0.05$). Interestingly, all light sheet imaged groups (one, five and ten) were comparable and not significantly different from the non-imaged (*Unexposed*) group (Fig. 5b; $P > 0.05$). When accounting for cell number, the DNA damage results persisted: Embryos that underwent repeated cycles of light sheet imaging had a significantly higher proportion of cells containing DNA damage when compared to embryos that were not imaged or underwent a single cycle of imaging (Fig. 5c; $P < 0.05$). These data show that light sheet imaging is capable of inducing DNA damage within the embryo, however, this requires multiple cycles of volumetric imaging. Furthermore, we observed that DNA damage for five and ten cycles of imaging was comparable and postulate that this may be due to this DNA repair mechanism reaching maximum capacity by five cycles of imaging^50^.

**Fig. 6:**
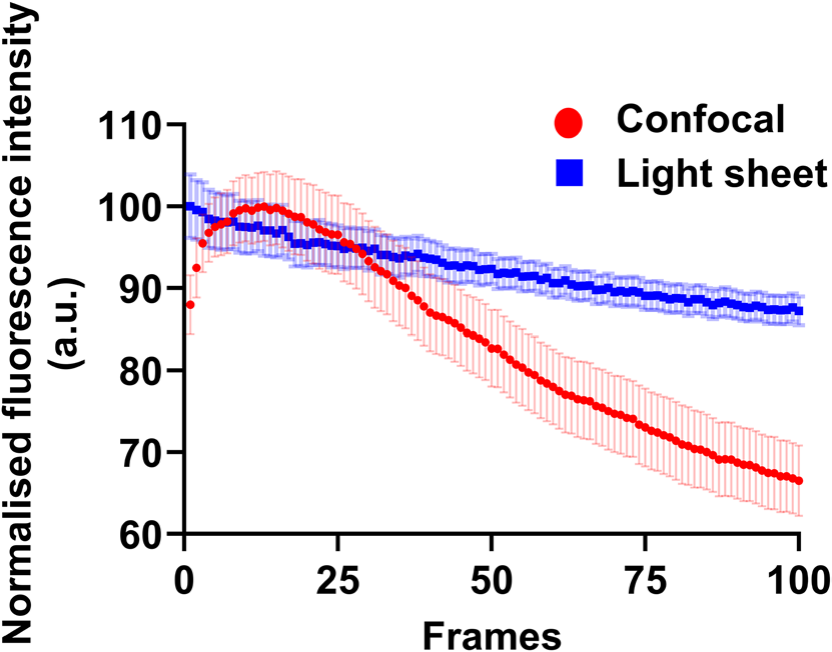
A semi-quantitative comparison of the photobleaching rate during confocal and light sheet imaging. Autofluorescence from blastocyst-stage embryos were recorded during confocal or light sheet imaging (both at 405 nm). To compare photobleaching, images at a single z-plane were acquired on both systems, using the same conditions as Fig. 2. Each embryo was imaged for 100 frames (n = 13 and 17 embryos for the light sheet and confocal groups, respectively). Data are presented as mean ± standard error of the mean.

We also evaluated the photobleaching rate of autofluorescence from embryos during confocal and light sheet imaging (405 nm excitation, Fig. 6). We found that the photobleaching rate was dramatically lower when embryos were imaged with light sheet microscopy compared to confocal microscopy (Fig. 6), providing evidence for the compatibility of light sheet microscopy for autofluorescence imaging. This is consistent with previous literature^23,51–54^ which focused on imaging using exogeneous fluorescent tags. Interestingly, we discovered an initial increase followed by a decrease in fluorescence intensity when using confocal microscopy (Fig. 6). This may be attributed to photobiological effects, altering underlying metabolic pathways or cellular structure^55–57^. Collectively, our findings show that confocal imaging is harmful to embryos, inducing levels of DNA damage that may not be compatible with subsequent development.

## Discussion

Optical imaging for developmental biology has gained prominence in recent times due to its ability to unveil complex biological processes with high spatial-temporal resolution. Optical imaging has been traditionally reliant on external fluorescent labeling. It is now transitioning toward label-free methods, utilising intrinsic autofluorescence emitted by endogenous molecules. An example is specific co-factors associated with cellular metabolism. In this study, we performed a comparative analysis between light sheet microscopy and confocal microscopy for autofluorescence imaging of preimplantation murine embryos. Our results provide evidence that light sheet microscopy is safer for embryo autofluorescence imaging than confocal microscopy.

First, we characterised the PSF for the two systems and found comparable lateral and axial resolutions for light sheet and confocal microscopy. Subsequently, we determined and optimised the imaging parameters for both systems to achieve comparable SNR for images of blastocyst-stage embryos. For a similar SNR, the image acquisition time was ten times longer with confocal microscopy, despite a larger step size (z-planes 2 $\upmu$m apart), when compared to light sheet microscopy (z-planes 1 $\upmu$m apart). Furthermore, in contrast to confocal microscopy, light sheet microscopy was able to generate a 3D image reconstruction that displays the autofluorescence signals for the entire volume of a blastocyst-stage embryo. We attribute this loss of signal associated with confocal microscopy to photobleaching from this point-scanning illumination approach. This observation aligns with existing literature that employed exogenous fluorophores^58,59^. This provides evidence demonstrating the efficiency and rapid image acquisition capability of light sheet microscopy, which agrees with previous literature^60^. We do note that light sheet microscopy may suffer from shadowing effects when imaging highly scattering samples. This was not an issue for the present work due to the relative transparency of embryos and their refractive index being comparable to water^61^.

Encouragingly, when assessing the impact of imaging, light sheet imaged and non-imaged embryos had comparable levels of DNA damage. Conversely, embryos imaged using the confocal microscope had a significantly higher level of DNA damage, persisting even after accounting for the total cell number within the embryo. It is important to note that any differences in nuclei shape, morphology, and size are likely to be attributed to the developmental stage of the embryo. It is at this stage that a fluid-filled cavity develops and continues to enlarge to a point where it takes up more than three quarters of the entire volume of the embryo. This expansion exerts a mechanical force that changes the shape and size of nuclei^62^. Consequently, any observed morphological variations in cell nuclei can be attributed to this developmental stage, which progresses as a continuum. Furthermore, in this study, DNA damage was assessed by identifying each nuclei containing $\gamma$H2AX (red) foci, and thus, the influence of nuclei shape and size on DNA damage is negligible and thus unlikely to confound our results. Furthermore, the low level of DNA damage in the light sheet imaged group is likely attributed to the thin, sheet-like geometry of the setup, which only illuminates the plane of interest. This contrasts with the point scanning method employed by confocal microscopy, where light traverses the entire volume of the embryo for each optical section^23^. Low levels of photodamage from light sheet imaging in this study are in agreement with existing literature^30^.

Interestingly, we found that five and ten rounds of repeated light sheet imaging resulted in a significant increase in DNA damage when compared to embryos that were imaged once or were not imaged. Based on our data, the threshold for inducing DNA damage necessitates more than one round of imaging. It would be of value to determine the minimum number of light sheet imaging cycles required to significantly increase DNA damage. We can conclude from our data that this lies somewhere between two and five cycles of volumetric imaging when using the imaging parameters described here. While the number of cycles required to induce elevated levels of DNA damage may be less than five, our study provides evidence that the limit of DNA damage able to be detected is reached with five rounds of light sheet imaging. We attribute this to the observation that ten repeated cycles did not result in a further increase in the level of DNA damage detected using $\gamma$H2AX immunohistochemistry. Further, we assessed photobleaching of autofluorescence in embryos. We found that autofluorescence intensity photobleached at a slower rate with light sheet compared to confocal imaging, highlighting the compatibility of light sheet for long-term monitoring of dynamic biological processes, such as changes in metabolic activity during preimplantation embryo development. Our findings on the rate of photobleaching with confocal and light sheet microscopy are in agreement with previous literature on other biological samples when using exogenous fluorescent tags^23,51–54^. We remark that the power level utilised in this study is both appropriate and sufficient to capture cellular autofluorescence at 405 nm (Fig. 2 and supplementary videos), providing evidence that the imaging conditions used here can yield valuable metrics for embryo metabolism and thus predicting viability. Collectively, these findings provide evidence that light sheet microscopy is a safe and efficient optical approach for embryo imaging, and affirms its potential as the preferred method for non-invasive imaging of embryos.

In this study, autofluorescence imaging of preimplantation murine embryos was conducted with an excitation wavelength of 405 nm. While our results affirm the safety of light sheet microscopy at this widely used wavelength, it would be interesting to investigate potential photodamage in embryos caused at other wavelengths in the UVA^10^ and visible ranges^63^ for metabolic studies using one-photon excitation. Our study focused on 405 nm illumination. A future study could also encompass the use of pulsed lasers for modalities such as fluorescence lifetime imaging, two-, and three-photon imaging. Two-photon fluorescence lifetime imaging in a point scanning geometry has shown merit for measuring differences in metabolism between embryos^12^. Moving to such multiphoton approaches generally improves excitation confinement and depth penetration, though at the expense of added complexity and cost. It would be interesting to explore how, in the two-photon approach, light sheet imaging compares to a point scanning approach. Such a study is outside the scope of the present work but could explore the pulsed laser parameters which are known to influence biological function^64,65^. Finally, we note that this study was conducted in a mouse model, necessitating additional research in other species to determine the generalisability of our findings to a broader biological context. Overall, our study shows that light-sheet imaging is safer than confocal microscopy for imaging embryo autofluorescence.

## Methods

### Light sheet microscopy

**Fig. 7:**
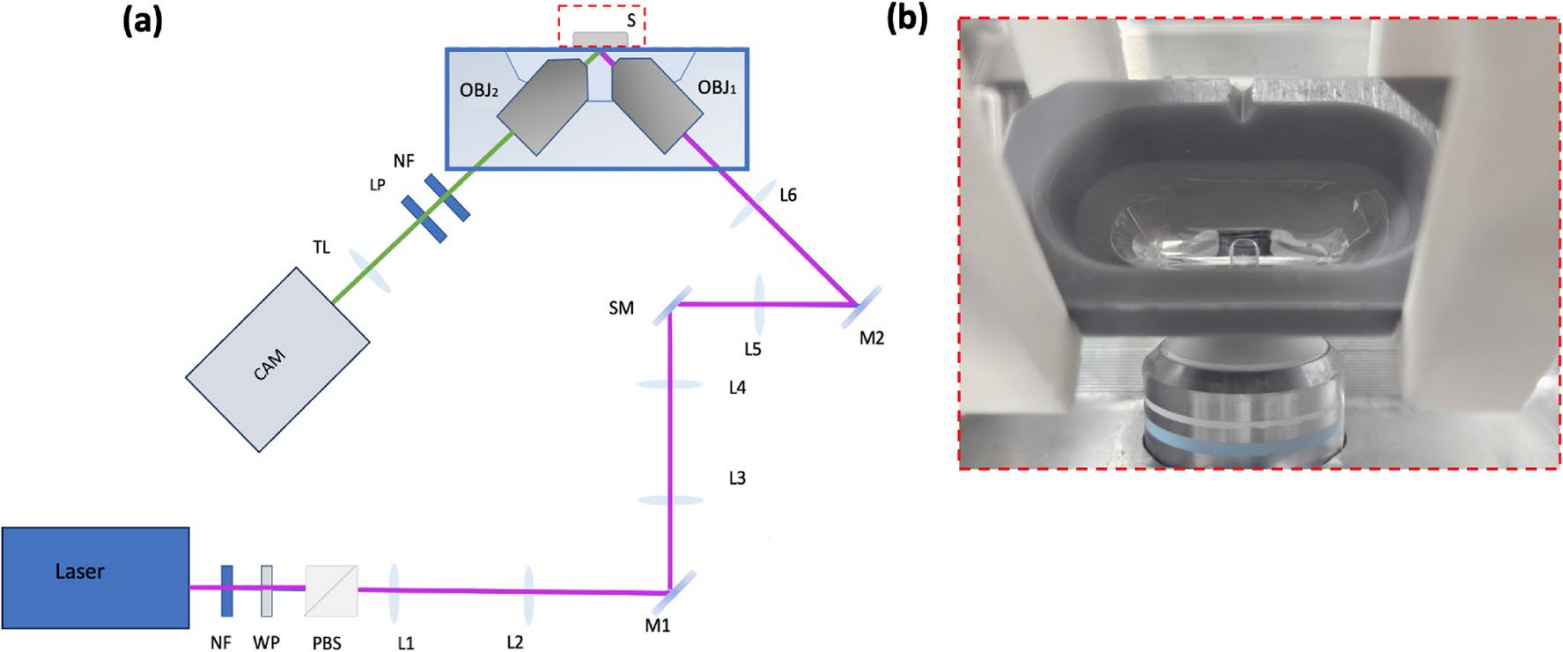
(**a**) Experimental setup for light-sheet measurements. Laser = 405 nm (Toptica iBeam Smart, 300 mW), NF = Notch Filter (407 nm), WP = half-waveplate, PBS = polarising beam splitter, L1 = 75 mm, L2 = 100 mm, L3 = 100 mm, L4 =100 mm, L5= 50 mm, L6 = 75 mm, M1–2= mirrors, SM = Scanning Mirror (Galvo, Thorlabs, NJ), $\hbox {OBJ}_\text {1}$ = 10X, Nikon and $\hbox {OBJ}_\text {2}$ = 16X, Nikon, S = sample holder, LP = long pass filter (405 nm), TL= Tube lens (400 mm), CAM = sCMOS camera (Iris 15, Teledyne Photometrics, AZ). (**b**) A top view of the sample holder lined with FEP film on top of the setup with a drop of imaging media, overlaid with paraffin oil, used for embryo imaging.

A schematic of the open-top light sheet setup is shown in Fig. 7a. A 405 nm continuous wave laser was used as the illumination source. The laser power was modulated by using a half-wave plate (WP) and a polarising beam splitter (PBS) and maintained at 3 mW at the sample. The beam was expanded by using a telescope (L1 and L2) and then relayed by lenses (L3–L6) and the illumination objective ($\hbox {OBJ}_\text {1}$). The light sheet was generated by a galvo scanner (SM) which rapidly scans the beam. Fluorescence was collected by the detection objective ($\hbox {OBJ}_\text {2}$) and relayed to the camera (CAM) by a tube lens (TL) after being filtered using a long pass filter (LP) and a notch filter (NF). A custom 3D-printed sample holder was mounted above the water reservoir and was attached to an XYZ translation stage to enable the sample holder to be lowered between the two objectives (see Fig. 7b). A 125 $\upmu$m thin membrane (FEP; Katco) with the same refractive index as water was placed into a groove in the sample holder and sealed with a biocompatible UV-cured glue applied to the bottom of the chamber at the interface between the chamber body and the membrane. The setup was also fitted with a controlled heating stage set to maintain the temperature within the chamber at 37 °C. In our setup, the imaging plane is oriented 45 degrees relative to the sample translation which is obtained using a high-resolution motorized linear actuator. A series of images were recorded with 1 $\upmu$m step size. To interpolate the images sequence into the physical coordinate space a custom Matlab software was used. The process of conversion to physical space has been described in detail in our previous work^66^. The optical power after the objective (sample plane) was measured to be $\sim 3$ mW, with an average signal-to-noise ratio (SNR) of 15.5 for the autofluorescence signals recorded with 405 nm excitation. We defined SNR as the mean signal intensity (Signal-background intensity) divided by the standard deviation of background intensity.

### Confocal microscopy

Confocal images were collected on an Olympus FV3000i laser scanning confocal microscope system, using 405 nm excitation. To obtain a comparable resolution a $20\times$ objective (Olympus UPLSAPO $20\times$) was used, with a numerical aperture of 0.75 and a working distance of 0.6 mm. The sample chamber was maintained at 37 °C at all times during imaging. Images were collected at a resolution of 1024 by 1024 pixels, with a spacing of 0.621 $\upmu$m giving a total field of view (FOV) of 636 $\upmu$m. Z-stacks were collected at 2 $\upmu$m intervals, covering a distance slightly larger than the embryos (average $\sim 50$ z-planes to include just above and below the whole embryo). Images were collected with a photomultiplier tube (PMT) setting of 500 V, and 40 $\upmu$s integration per pixel. The optical power at the objective was measured to be $\sim 0.345$ mW, with an average SNR of 15.7 for the autofluorescence signals recorded with 405 nm excitation.

### Preparation of phantoms for light sheet and confocal imaging

In order to characterise the systems’ point spread function (PSF) phantoms made up of 200 nm diameter green fluorescent microspheres mixed with 1$\%$ agarose were prepared. The samples were pipetted into a 3D-printed sample holder for light sheet imaging (Fig. 7b) and a 35 mm glass-bottom dish (Ibidi, Martinsried, Planegg, Germany) for confocal imaging. The parameters used for phantom imaging on both systems were the same used for embryo imaging, which gives an SNR value of 15.5 for light sheet and 15.7 for confocal microscope (Fig. 2). However, when the same settings for embryo imaging were used for phantom imaging using confocal microscope (where spatial resolution was 1 pixel = 0.621 $\upmu$m), the 200 nm beads were effectively limited to a single pixel (Supplementary Fig. S1) and consequently, the details of PSF could not be accurately resolved due to under-sampling. To address this, we implemented Nyquist’s theorem by increasing the optical zoom while maintaining other parameters equal to that used for embryo imaging (such as imaging dish, laser power, and objective lens), which increases the spatial resolution from 0.621 to 0.13 $\upmu$m. This Nyquist-optimised parameter was used for PSF quantification of phantoms as shown in Fig. 1.

### Animal ethics and embryo collection

Female (21–23 days old) and male (6–8 weeks old) CBA x C57BL6 first filial (F1) generation mice were obtained from Laboratory Animal Services (University of Adelaide, Australia) and maintained on a 12 h light:12 h dark cycle with rodent chow and water provided ad libitum. All animal studies associated with this project were approved by the University of Adelaide’s Animal Ethics Committee (M-2019-097) and were conducted in accordance with the Australian Code of Practice for the Care and Use of Animals for Scientific Purposes. The studies are reported in accordance with ARRIVE guidelines^67^. Mice were hormonally stimulated to harvest embryos as previously described^9^. Briefly, female mice were administered intraperitoneally (i.p.) with 5 IU of equine chorionic gonadotrophin (eCG), followed by 5 IU of human chorionic gonadotrophin (hCG) 48 h later. At 13 h post-hCG, male mice of proven fertility were culled by cervical dislocation and the epididymis and vas deferens harvested. Spermatozoa were released from the corpus and caudal region of the epididymis as well as the vas deferens by blunt dissection into pre-warmed Research Fertilisation medium (ARTLab Solutions, Australia) and allowed to capacitate for 45 min in a humidified incubator at $37\,^{\circ }$C with 5% $\hbox {O}_\text {2}$, 6% $\hbox {CO}_\text {2}$, balanced in air. Female mice were culled by cervical dislocation at 14 h post-hCG and oviducts harvested. Ovulated cumulus-oocyte complexes (COCs) were released from the oviducts by gently puncturing the ampulla with a 29-gauge needle. At 14 h post-hCG, ovulated COCs and capacitated spermatozoa were co-incubated in Research Fertilisation media for 4 h in a humidified incubator at $37\,^{\circ }$C with 5% $\hbox {O}_\text {2}$, 6% $\hbox {CO}_\text {2}$, balanced in air. Resulting presumptive zygotes were transferred into Research Cleave medium (in groups of 10; 2 $\upmu$l per embryo) and allowed to develop to the blastocyst stage.

### Sample preparation for imaging using light sheet and confocal microscopy

Blastocyst-stage embryos were collected at 96 h post-IVF and allocated into either a dedicated purpose-built imaging chamber, lined with a 127 $\upmu$m FEP film for light sheet imaging (as described in Fig.7b), or a 35 mm glass bottom IBIDI dish for confocal imaging. For light sheet imaging, embryos were imaged in a 20 $\upmu$l drop of Research Wash medium (ARTLab Solutions, Australia) overlaid with paraffin oil. A similar setup was implemented for confocal imaging, with the exception that embryos were imaged in a 10 $\upmu$l drop of Research Wash medium. The Research Wash medium provides a physiological-pH buffered medium for live embryo imaging. All imaging was performed with the temperature of media maintained at $37\,^{\circ }$C. All embryos following imaging were returned to culture in the incubator for an additional 30 min prior to fixation for DNA damage analysis, to allow for appropriate activation of DNA damage response, i.e. recruitment of histone $\gamma$H2AX to the site of DNA double-strand breaks^43^.

### Phototoxicity and photobleaching assessment following light sheet and confocal microscopy

To assess phototoxicity, blastocyst-stage embryos were kept in the dark (unexposed) or imaged using either confocal or light sheet microscopy. A separate cohort of embryos was also included to assess the impact of repeated imaging cycles with light sheet microscopy: one, five, or ten rounds of volumetric imaging using light sheet microscopy that corresponds to a total imaging duration of three, fifteen, or thirty minutes, respectively. This was compared to confocal microscopy, which required approximately thirty minutes to acquire a single z-stack. For the assessment of photobleaching, a total of 100 frames were captured at a single z-plane for blastocyst-stage embryos. A region of interest within the inner cell mass of blastocyst-stage embryo was used to calculate the changes in autofluorescence intensity across all 100 frames.

### Immunohistochemistry for DNA damage

All immunohistostaining procedures were carried out at room temperature. Immunofluorescence for phosphorylated gamma-H2AX ($\gamma$H2AX) was used to assess for double-stranded DNA breaks as previously described^68^. Briefly, blastocyst-stage embryos were fixed for 30 min in 400 $\upmu$l of 4% paraformaldehyde (PFA) diluted in phosphate buffer saline (PBS). After fixation, embryos were washed with 200 $\upmu$l of 0.3 mg/ml polyvinyl alcohol in PBS (PBV) and permeabilised for 30 min in 0.25% Triton-X diluted in PBS. To prevent non-specific binding, embryos were blocked for 1 h in 2% bovine serum albumin (BSA) diluted in PBS. Embryos were then incubated for 1 h with anti-yH2AX rabbit monoclonal Alexa Fluor 488-conjugated primary antibody (Ser139, 20E3, Cell Signaling Technology, Danvers, MA, USA) at 1:200 dilution in 1% BSA in PBS. A negative control without primary antibody in an otherwise complete 1% BSA/PBS solution was also included. Following incubation, embryos were washed with PBV three times before incubation for 1 h in the dark with an anti-rabbit Alexa Fluor 594-conjugated secondary antibody (Life Technologies, Carlsbad, CA, USA) at 1:400 dilution in 1% BSA in PBS. Embryos were also counterstained with 3 mM of 4,6-diamidino-2-phenylindole (DAPI) to visualise nuclei. After secondary antibody incubation, embryos were washed with PBV three times before being mounted on glass slides with secure-seal spacers (0.12 mm apart) in 5 $\upmu$l PBV and sealed with a 22 mm $\times$ 22mm glass coverslip prior to imaging.

### Image acquisition and analysis of $\gamma$H2AX immunostaining

All images of $\gamma$H2AX immunostaining were captured on an Olympus FV3000i laser scanning confocal microscope (Olympus, Tokyo, Japan). Images were collected with a 60x immersion oil-compatible objective (Olympus, NA = 1.4). Images were captured at 2-$\upmu \hbox {m}$ intervals through the entire embryo and a final z-stack was generated for each embryo. Samples were excited at a laser wavelength of 405 nm (emission wavelength detection range: 430–470 nm) for DAPI and 594 nm (emission detection range: 499–520 nm) for $\gamma$H2AX. Image analysis was performed using ImageJ for Windows 10 (Fiji, MD, USA). For image analysis of $\gamma$H2AX, z-stack images of DAPI and $\gamma$H2AX channels were first merged, and then the number of nuclei containing $\gamma$H2AX-positive foci was counted manually.

### Statistical analysis

Statistical analysis for DNA damage was performed using GraphPad Prism version 9 for Windows 10 (GraphPad Holdings LLC, CA, USA). Data were checked for normality and subjected to appropriate statistical testing as described in the figure legend. For example, as data in Fig. 4c did not follow a normal (Gaussian) distribution, a Kruskal-Wallis with Dunn’s multiple comparison test was used for statistical analysis, as opposed to a one-way ANOVA with Tukey’s multiple comparison test for normally distributed data (Fig. 4a,b). Statistical significant differences were set at $P < 0.05$.

## Supplementary Information

Supplementary Information.

## Data Availability

Data associated with this research are available and can be obtained by contacting the corresponding author upon reasonable request.
