# Supplementary material for: Quantifying DNA damage following light sheet and confocal imaging of the mammalian embryo
Source: Sci Rep. 2024 Sep 5;14:20760. doi: 10.1038/s41598-024-71443-x (PMC11377761; doi:10.1038/s41598-024-71443-x)
Supplement: Supplementary file 1 — Supplementary Information. [file 41598_2024_71443_MOESM1_ESM.pdf]

Supplementary Information

1 S1 - Determining the point spread function for confocal microscopy

In this section, we investigate the point spread function using the imaging parameters on the FV3000 confocal microscopy, which were employed for embryo imaging. Phantoms (200 nm fluorescence beads) were generated as described in the methods section of the main manuscript. Figure 6 shows the maximum intensity profile for a single bead and the line profile through it in the XY and YZ planes. As the imaging parameters generated images with a spatial resolution of 0.621 μm per pixel, the 200 nm beads were effectively limited to a single pixel, and consequently, the details of PSF could not be accurately resolved due to under-sampling. To overcome this, we implemented Nyquist's theorem by increasing the optical zoom on the confocal system while maintaining all other parameters identical to that used for embryo imaging, increasing the spatial resolution from 0.621 μm to 0.13 μm per pixel. This Nyquist-optimised imaging parameter was used for PSF quantification of phantoms, as shown in Figure 1 within the main manuscript.

2 S2 - 3D reconstruction of embryo using either confocal or light sheet microscopy

In this section, we present videos demonstrating 3D image reconstructions of volumetric images acquired for an embryo using either confocal or light sheet microscopy. Interestingly, volumetric images obtained with confocal microscopy had a limited imaging capability for this sample, resulting in a loss of approximately half the volume of the embryo (Video 1; Confocal). In contrast, light sheet microscopy effectively captured the entire volume of a blastocyst-stage embryo, allowing for true 3D image reconstruction (Video 2; Light sheet). File depository for Video 1 (Confocal) and 2 (Light sheet) can be found here:

https://universityofadelaide.box.com/s/cpkvenoinslusivmpqnxnu4tdl4f15va

3 S3a and S3b- Quantification of DNA damage within blastocyst-stage embryos following confocal or light sheet microscopy

This section presents quantitative data on the total number of cells within the blastocyst-stage embryos following imaging and the absolute count of nuclei con-

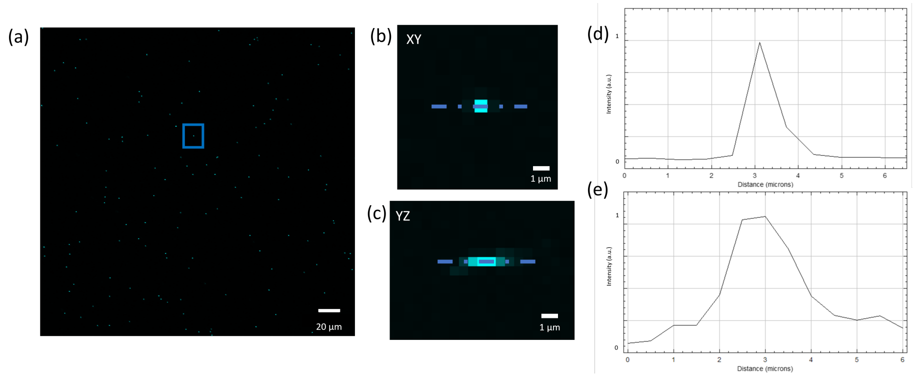

Figure 1: (a) Maximum intensity projection of 200 nm diameter fluorescence beads embedded in agarose imaged using confocal microscopy. Imaging parameters were the same used for embryo imaging. (b) and (c) show the intensity projection in xy and yz plane of a single bead as outlined in (a). (d) and (e) show the corresponding line profile across the bead in xy and yz plane respectively.

taining γH2AX-positive nuclei. These data were utilised to calculate the percentage of DNA damage, representing a proportion of cells containing γH2AX foci, as detailed in Figure 5 in the main manuscript.

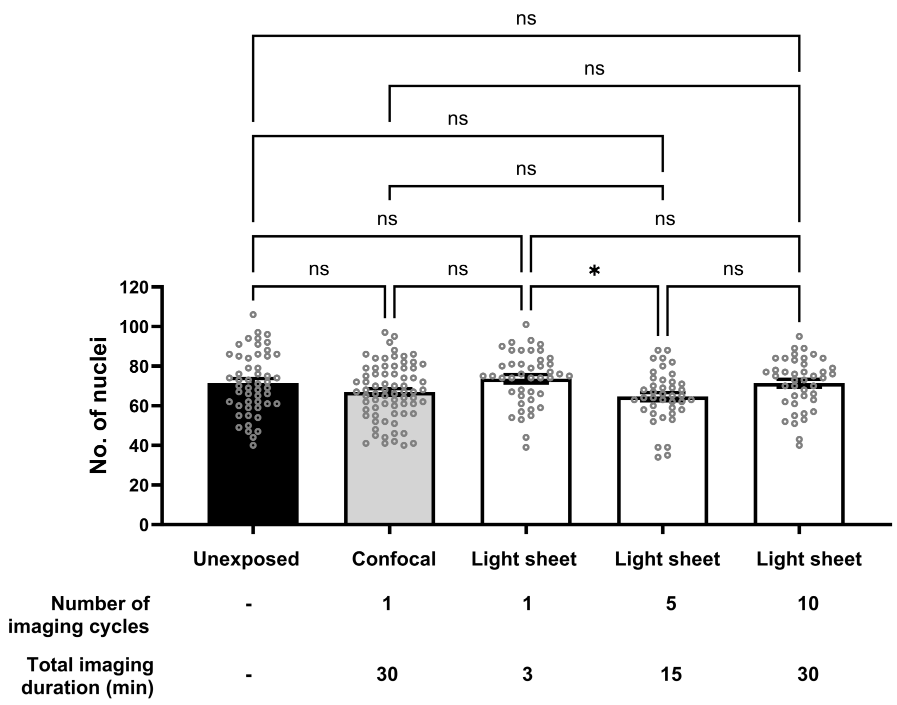

Figure 2: DC: New FIGURE(a) Number of cells within blastocyst-stage embryos following imaging with confocal or light sheet microscopy. Blastocyst-stage embryos were either kept in the dark (unexposed), imaged using confocal microscopy (one z-stack) or subjected to increasing rounds of imaging using light sheet microscopy (one, five or ten z-stacks). The total imaging duration to acquire these z-stack(s) is shown. Following imaging, embryos were returned to culture for 30 min prior to fixation. DNA damage and cell nuclei were identified via γH2AX immunohistochemistry and DAPI staining, respectively. Data are presented as mean $\pm$ SEM, n = 43-72 embryos, from 3 independent experimental replicates, analysed using Kruskal-Wallis with Dunn's multiple comparison test. $*P < 0.05$; $***P < 0.001$; $****P < 0.0001$

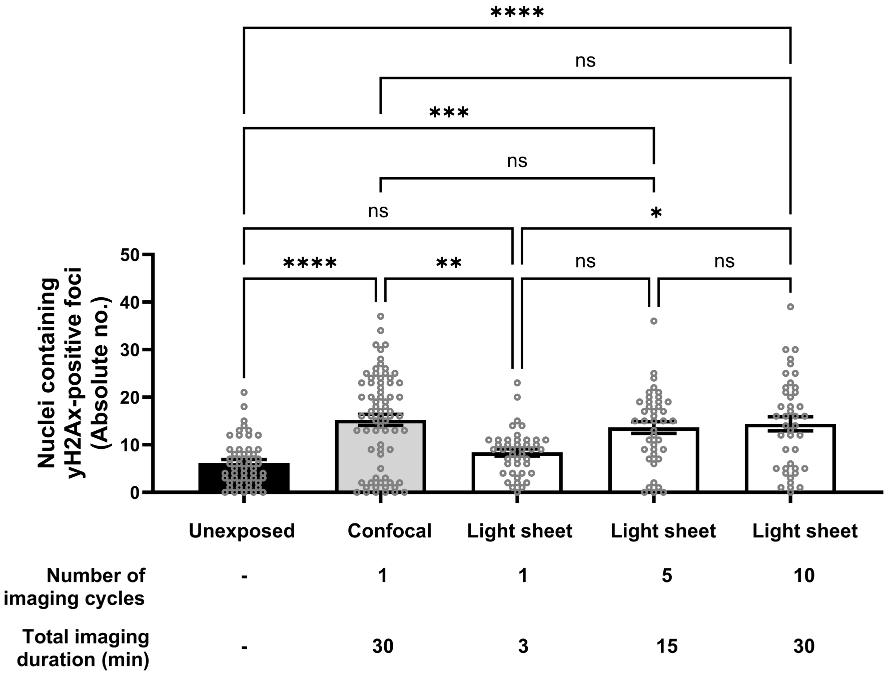

Figure 3: DC: New FIGURE(b) Number of cells containing γH2AX-positive foci. Blastocyst-stage embryos were either kept in the dark (unexposed), imaged using confocal microscopy (one z-stack) or subjected to increasing rounds of imaging using light sheet microscopy (one, five or ten z-stacks). The total imaging duration to acquire these z-stack(s) is shown. Following imaging, embryos were returned to culture for 30 min prior to fixation. DNA damage and cell nuclei were identified via γH2AX immunohistochemistry and DAPI staining, respectively. Data are presented as mean ± SEM, n = 43-72 embryos, from 3 independent experimental replicates, analysed using Kruskal-Wallis with Dunn's multiple comparison test. *$P < 0.05$; ***$P < 0.001$; ****$P < 0.0001$

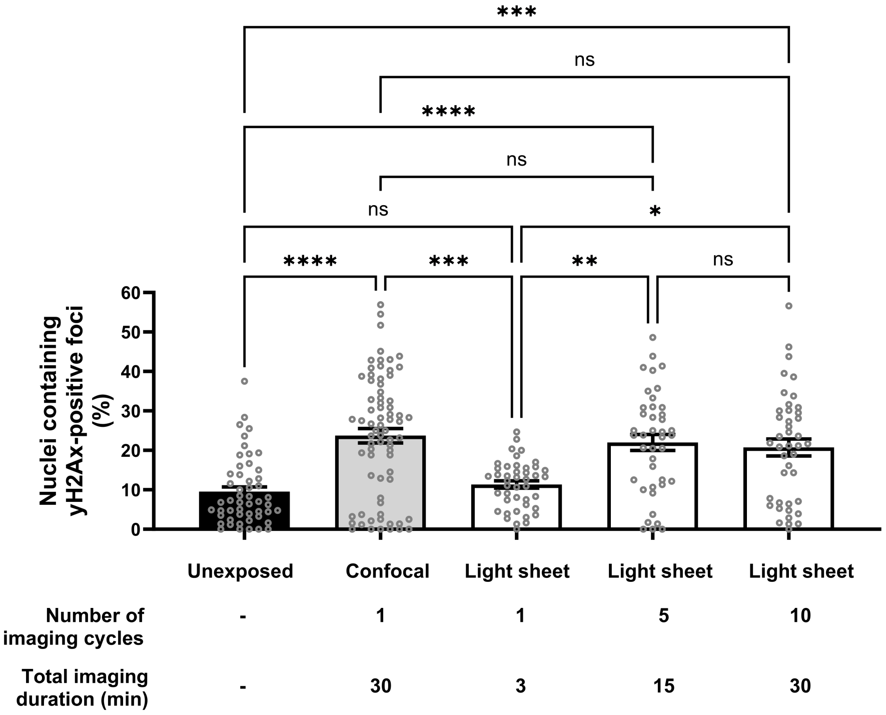

Figure 4: DC: New FIGURE(c) Percentage of cells containing γH2AX-positive foci. Blastocyst-stage embryos were either kept in the dark (unexposed), imaged using confocal microscopy (one z-stack) or subjected to increasing rounds of imaging using light sheet microscopy (one, five or ten z-stacks). The total imaging duration to acquire these z-stack(s) is shown. Following imaging, embryos were returned to culture for 30 min prior to fixation. DNA damage and cell nuclei were identified via γH2AX immunohistochemistry and DAPI staining, respectively. Data are presented as mean $\pm$ SEM, n = 43-72 embryos, from 3 independent experimental replicates, analysed using Kruskal-Wallis with Dunn's multiple comparison test. $*P < 0.05$; $***P < 0.001$; $****P < 0.0001$

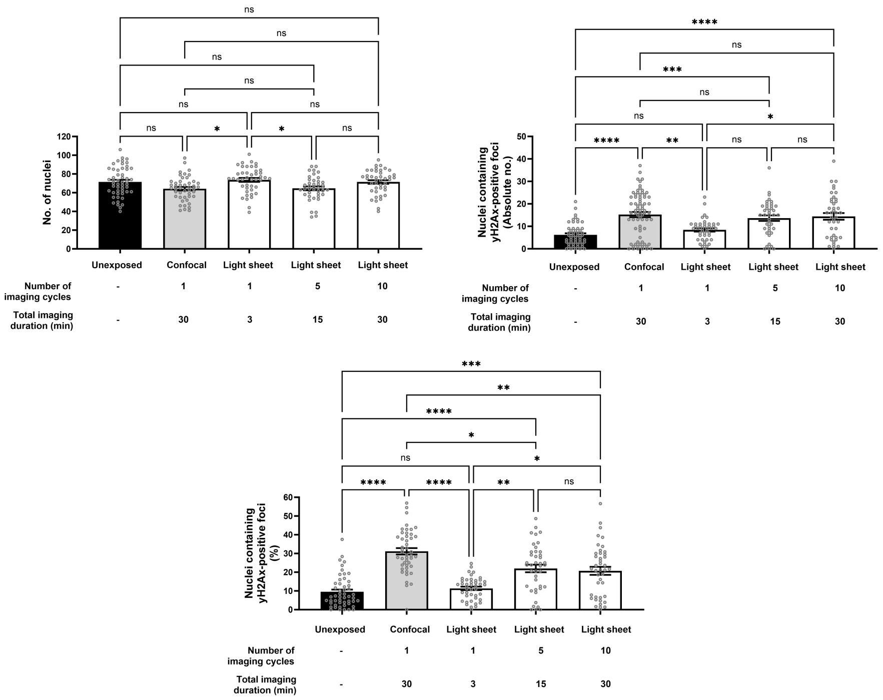

Figure 5: DC: New FIGURE-VerticalCombined all new figures

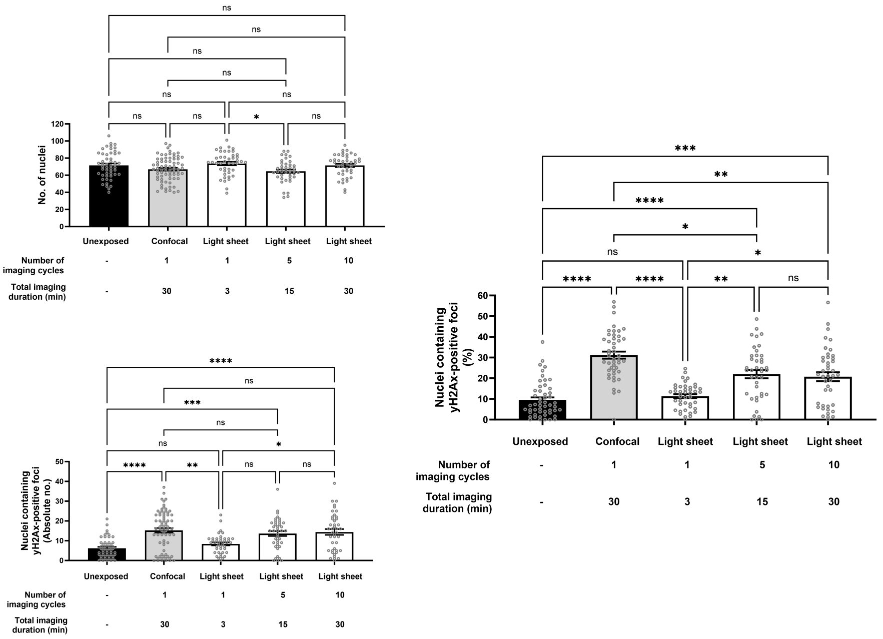

Figure 6: DC: New FIGURE-HorizontalCombined all new figures
